# Virus-Host and CRISPR Dynamics in Archaea-Dominated Hypersaline Lake Tyrrell, Victoria, Australia

**DOI:** 10.1155/2013/370871

**Published:** 2013-06-18

**Authors:** Joanne B. Emerson, Karen Andrade, Brian C. Thomas, Anders Norman, Eric E. Allen, Karla B. Heidelberg, Jillian F. Banfield

**Affiliations:** ^1^Department of Earth and Planetary Science, University of California, Berkeley, 307 McCone Hall, Berkeley, CA 94720-4767, USA; ^2^Cooperative Institute for Research in Environmental Sciences, University of Colorado, Boulder, CO, USA; ^3^Department of Environmental Science, Policy, and Management, University of California, Berkeley, 54 Mulford Hall, Berkeley, CA 94720, USA; ^4^Department of Biology, University of Copenhagen, Copenhagen, Denmark; ^5^Marine Biology Research Division, Scripps Institution of Oceanography, La Jolla, CA, USA; ^6^Division of Biological Sciences, University of California, San Diego, La Jolla, CA 92093-0202, USA; ^7^Department of Biological Sciences, University of Southern California, Los Angeles, CA 90089, USA

## Abstract

The study of natural archaeal assemblages requires community context, namely, a concurrent assessment of the dynamics of archaeal, bacterial, and viral populations. Here, we use filter size-resolved metagenomic analyses to report the dynamics of 101 archaeal and bacterial OTUs and 140 viral populations across 17 samples collected over different timescales from 2007–2010 from Australian hypersaline Lake Tyrrell (LT). All samples were dominated by Archaea (75–95%). Archaeal, bacterial, and viral populations were found to be dynamic on timescales of months to years, and different viral assemblages were present in planktonic, relative to host-associated (active and provirus) size fractions. Analyses of clustered regularly interspaced short palindromic repeat (CRISPR) regions indicate that both rare and abundant viruses were targeted, primarily by lower abundance hosts. Although very few spacers had hits to the NCBI nr database or to the 140 LT viral populations, 21% had hits to unassembled LT viral concentrate reads. This suggests local adaptation to LT-specific viruses and/or undersampling of haloviral assemblages in public databases, along with successful CRISPR-mediated maintenance of viral populations at abundances low enough to preclude genomic assembly. This is the first metagenomic report evaluating widespread archaeal dynamics at the population level on short timescales in a hypersaline system.

## 1. Introduction

 As the most abundant and ubiquitous biological entities, viruses influence host mortality and community structure, food web dynamics, and geochemical cycles [1, 2]. In order to better characterize the potential influence that viruses have on archaeal evolution and ecology, it is important to understand the coupled dynamics of viruses and their archaeal hosts in natural systems. Although previous studies have demonstrated dynamics in virus-host populations, most of these studies have focused on bacterial hosts, often restricted to targeted groups of virus-host pairs, and little is known about archaeal virus-host dynamics in natural systems. 

 Community-scale virus-host analyses have often been based on low-resolution measurements of the whole community, relying on techniques such as denaturing gradient gel electrophoresis (DGGE), pulsed-field gel electrophoresis (PFGE), and microscopic counts (e.g., [3–5]). One exception is a study that examined viral and microbial dynamics through single read-based metagenomic analyses in four aquatic environments, including an archaea-dominated hypersaline crystallizer pond [6]. In that work, it was proposed that microorganisms and viruses persisted over time at the level of individual taxa (species) but were highly dynamic at the genotype (strain) level. However, in a reanalysis of some of those data by our group using metagenomic assembly, we concluded that viruses were actually dynamic at the population (taxon) level in that system [7]. This result suggests that further analyses are necessary to determine whether archaeal populations tend to be dynamic or stable in hypersaline systems on short timescales.

 Of the relatively few metagenomic analyses of virus-host dynamics that have been reported, several have considered the clustered regularly interspaced short palindromic repeat (CRISPR) system, which provides an opportunity to study hosts' responses to viral predation and to link viruses to hosts [8–12]. The CRISPR system is a genomic region in nearly all archaea and some bacteria, and CRISPRs (at least in all systems that have been biochemically characterized to date) have been shown to confer adaptive immunity to viruses and/or other mobile genetic elements through nucleotide sequence identity between the host CRISPR system and invading nucleic acids [13, 14]. The hallmarks of CRISPR regions are spacers, generally derived from foreign nucleic acids, including plasmid and viral DNA, and short palindromic repeat sequences between each spacer (reviewed in [15, 16]). Different strains of the same species can have highly divergent CRISPR regions (e.g., [17]). A highly genomically resolved study of virus-host dynamics in an archaea-dominated acid-mine drainage system demonstrated that only the most recently acquired CRISPR spacers matched coexisting viruses and showed that viruses rapidly recombined to evade CRISPR targeting, indicating that community stability was achieved by the rapid coevolution of host resistance to viruses and viral resistance to the host CRISPR system [9]. Archaeal CRISPR dynamics have also been investigated in *Sulfolobus islandicus* populations [18, 19], indicating clear biogeography of viral populations and adaptation of CRISPR sequences to local viral populations. Whether similar dynamics occur in archaea-dominated hypersaline systems is not well understood.

 Previously, our group tracked the dynamics of 35 viral populations in eight viral concentrates (representing the 30 kDa–0.1 *μ*m size fraction) collected during three summers from archaea-dominated hypersaline Lake Tyrrell (LT), Victoria, Australia [7]. We demonstrated that viruses in the LT system were generally stable on the timescale of days and dynamic over years. In this study, we sought to expand our analyses to include LT viruses from metagenomic libraries generated from 0.1, 0.8, and 3.0 *μ*m filters, potentially including proviruses, viruses larger than 0.1 *μ*m, actively infecting viruses, and viruses otherwise retained on the filters. This allowed us to increase the temporal and spatial scope of our study to 17 samples, including four winter samples from which viral concentrate DNA was not sequenced. To give context to the current and previous viral analyses from LT and to test the prevailing theory that microbial taxa are stable at the species level in archaea-dominated hypersaline systems (presented in [6]), in this study we also characterized archaeal and bacterial dynamics through 16S rRNA gene analyses, and we used CRISPR analyses to assist in the interpretation of the results.

## 2. Materials and Methods

### 2.1. Sample Collection and Preparation

 Sample collection, DNA extraction, and sequencing methods have been described previously [7, 20, 21]. Briefly, 10 L surface water samples were collected from LT and sequentially filtered through 20, 3.0, 0.8, and 0.1 *μ*m filters. Post-0.1 *μ*m filtrates were concentrated through tangential flow filtration and retained for viral DNA extraction. Viral concentrates and 0.1, 0.8, and 3.0 *μ*m filters were retained for each sample, and sequencing was undertaken for different size ranges, depending on the sample (Table 1). Sample names include the month (J for January, A for August), year, site (A or B, ~300 m apart), and time point if the sample was part of a days-scale time series (e.g., t1, t2, etc.). Where necessary, the size fraction is also indicated in the sample name (3.0, 0.8, or 0.1 for filter size in *μ*m, or VC for viral concentrate). Sites A and B are isolated pools in the summer (January samples) but continuous with the lake in the winter (August samples). GPS coordinates for sites A and B are 35° 19′ 09.6′′ S, 142° 47′ 59.7′′ E and 35° 19′ 18.71′′ S, 142° 48′ 4.23′′ E, respectively.

### 2.2. Recovery of 140 Viral Contigs >10 kb and Detection of Viruses in Each Sample

 In addition to the 35 LT viral and virus-like (meaning virus or plasmid) populations that we described previously [7], we incorporated all contigs >10 kb from a new IDBA_UD [22] assembly of the six Illumina-sequenced viral concentrate samples (default parameters). We also sought to include as many assembled viral sequences as possible from libraries from larger size-fraction filters. To do that, we first attempted to assemble reads from all Illumina-sequenced filter samples and reads from at least one 0.8 *μ*m filter per sample (regardless of sequencing type), using IDBA_UD with default parameters [22] for Illumina-sequenced samples and gsAssembler [23] with default parameters for 454-sequenced samples. The assemblies were generally fragmentary, and, as such, no viral contigs larger than 10 kb were identified from most assemblies. However, we recovered viral contigs >10 kb from metagenomic assemblies of all 0.1 *μ*m filters from January 2010, identified through annotation that could be confidently assigned to viruses or proviruses (e.g., contigs that included viral capsid proteins, tail proteins, and/or terminases). Annotation parameters were the same as those described previously [7]. We used BLASTn to identify duplicate viral sequences across assemblies and samples, and we removed any contigs that shared >2 kb at >95% nt identity (all contigs that were removed actually shared ≥99% nt identity because no contigs shared >2 kb at 88–98% nt identity). The remaining 140 viruses share up to 2 kb at up to 87% nt identity, with some smaller shared regions at higher identity.

 To determine whether or not a given virus was present in a given sample, we used the 140 viral contig sequences >10 kb as references for fragment recruitment (i.e., recruitment of Illumina sequencing reads or equivalent 100 bp read fragments from other sequencing technologies, as described in [7]), using the Burrows-Wheeler Aligner (bwa) with default parameters [24]. We required at least 1x read coverage across at least 50% of a given reference contig sequence for detection. For hierarchical clustering (Pearson correlation, average linkage clustering), the number of reads that mapped to a given viral contig sequence in a given sample was normalized by the length of the viral sequence and the number of reads in the sample, as described previously [7].

### 2.3. Generation of 16S rRNA Gene Data and Calculations of Host Relative Abundance

 To generate a reference database of 16S rRNA gene sequences, we used the EMIRGE algorithm [25] to reconstruct near full-length 16S rRNA genes from Illumina metagenomic data. All 0.1 and 0.8 *μ*m filters, from which DNA was Illumina sequenced and from which viral concentrate DNA was also sequenced from the same sample, underwent EMIRGE analysis in order to generate a reference 16S rRNA gene database for the LT system. The following filter sample metagenomes were EMIRGE analyzed: 2007At1 (0.8 *μ*m), 2010Bt3 (0.8 *μ*m), 2010Bt1 (0.1 *μ*m), 2010Bt2 (0.1 *μ*m), 2010Bt3 (0.1 *μ*m), and 2010A (0.1 *μ*m). We clustered all EMIRGE-generated 16S rRNA gene sequences at 97% nt identity, using UCLUST [26], resulting in a database of 101 16S rRNA gene sequences. Taxonomy was assigned to these OTUs, using the SILVA Incremental Aligner (SINA) [27] on the SILVA website [28, 29]. Using bwa with default parameters [24], we mapped metagenomic reads (split into 100 bp lengths to limit biases associated with different sequencing technologies, as described above and in [7]) from all filter samples to the reference database of 101 OTUs, in order to generate relative abundance estimates for each OTU across samples. For a given OTU to be detected in a given library, we required ≥1x coverage across ≥70% of the length of the EMIRGE-generated 16S rRNA gene sequence. In order to account for differences in sequencing throughput, we used relative abundances; that is, we estimated the percent abundance of each OTU in each sample as the number of reads that mapped to that OTU divided by the total number of reads that mapped to any OTU in that sample times 100. Those relative abundances were used for hierarchical clustering (Pearson correlation, average linkage clustering) and appear in Table S1 (see Supplementary Material available online at http://dx.doi.org/10.1155/2013/370871).

 These sequences (140 viral contigs >10 kb and 101 16S rRNA gene sequences) have been submitted to NCBI under BioProject accession no. PRJNA81851. 

### 2.4. Correlation with CRISPR Spacers

 We used Crass [30] to identify clustered regularly interspaced short palindromic repeat (CRISPR) repeat and spacer sequences in each sample. For this analysis, we considered all sequenced filter sizes for a given water sample together (i.e., 0.1, 0.8, and 3.0 *μ*m filters). Using BLASTn with an *E*-value cutoff of 1*e* − 10, we assessed the number of CRISPR spacers from each sample that matched (1) any of the 140 viral contig sequences >10 kb, (2) reads from viral concentrates collected from the same sample (applicable only to the eight samples from which viral concentrates were sequenced), and (3) reads from viral concentrates collected from other samples. 

## 3. Results and Discussion

### 3.1. Relative Abundances of Viral Populations across Size Fractions

 Contigs >10 kb from 105 new viruses were reconstructed, increasing the number of genomically characterized viruses from Lake Tyrrell (LT), Victoria, Australia, from 35 to 140. The 140 contigs, including seven previously reported complete genomes, range in size from 10,050 to 93,283 bp. We analyzed the relative abundances of these 140 viral genotypes in metagenomic libraries from viral concentrates and filter size fractions (0.1–0.8, 0.8–3.0, and 3.0–20 *μ*m) across time and between locations in LT. As few as 42 and as many as 116 viruses were detected in a given sample (any size fraction), and more viruses were detected in samples from which viral concentrates were sequenced. Though we acknowledge that a comparison of 16S rRNA gene microbial OTUs to >10 kb viral contig OTUs is an imperfect proxy for comparing the diversity of these groups, we find approximately 10 viral populations in the viral concentrate size fraction per host OTU (any filter size fraction) in most samples, suggesting that planktonic virus diversity and host diversity scale approximately with abundance, which has previously been established to be approximately 10 : 1 in most environments (e.g., [31]). 

**Figure 1:**
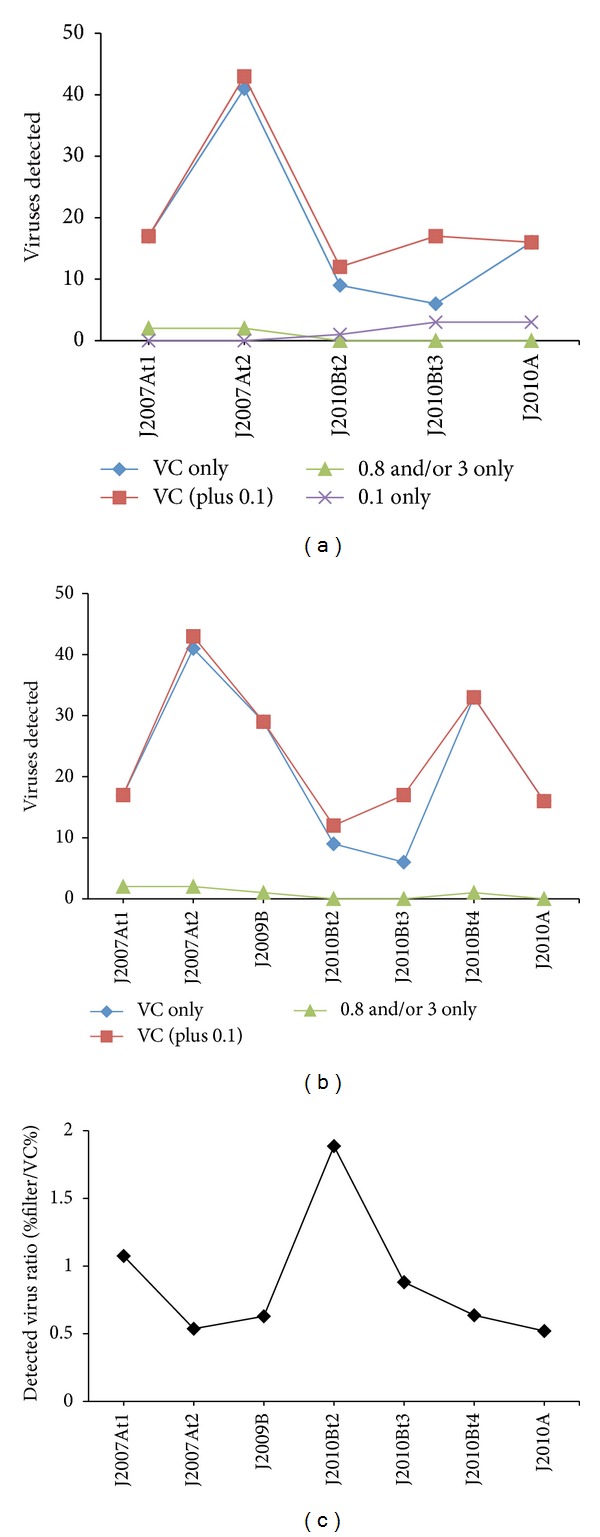


 In an attempt to distinguish among free (planktonic) viruses physically trapped on filters and host-associated viruses (i.e., active viruses and/or proviruses), we assumed that the 0.8 and 3.0 *μ*m filter pore sizes would be too large to retain significant numbers of viral particles and should predominantly include host-associated viruses. We assumed that the 0.1 *μ*m filters could retain both host-associated and planktonic viruses and that the viral concentrates (30 kDa–0.1 *μ*m size fraction) should generally exclude host cells and be dominated by planktonic viruses. Therefore, we considered viral detection in libraries from three filter size groups separately: (1) viral concentrates, (2) 0.1 *μ*m filters, and (3) 0.8 and/or 3.0 *μ*m filters. For five samples from which at least one library from each of those groups was sequenced, the number of viruses detected only in the viral concentrates was always higher than the number detected only on filters (Figure 1(a)). That trend is robust to the addition of two more samples, from which libraries from viral concentrates and at least one 0.8 and/or 3.0 *μ*m filter sample were sequenced (Figure 1(b)). Although we detected more unique viruses in the viral concentrates, the ratio of total viruses detected on 0.8 or 3.0 *μ*m filters, relative to total viruses detected in the viral concentrates, tended to be approximately equal (Figure 1(c)), meaning that the richness of viruses in viral concentrates tended to be similar to viral richness in the host-associated size fractions.

**Figure 2:**
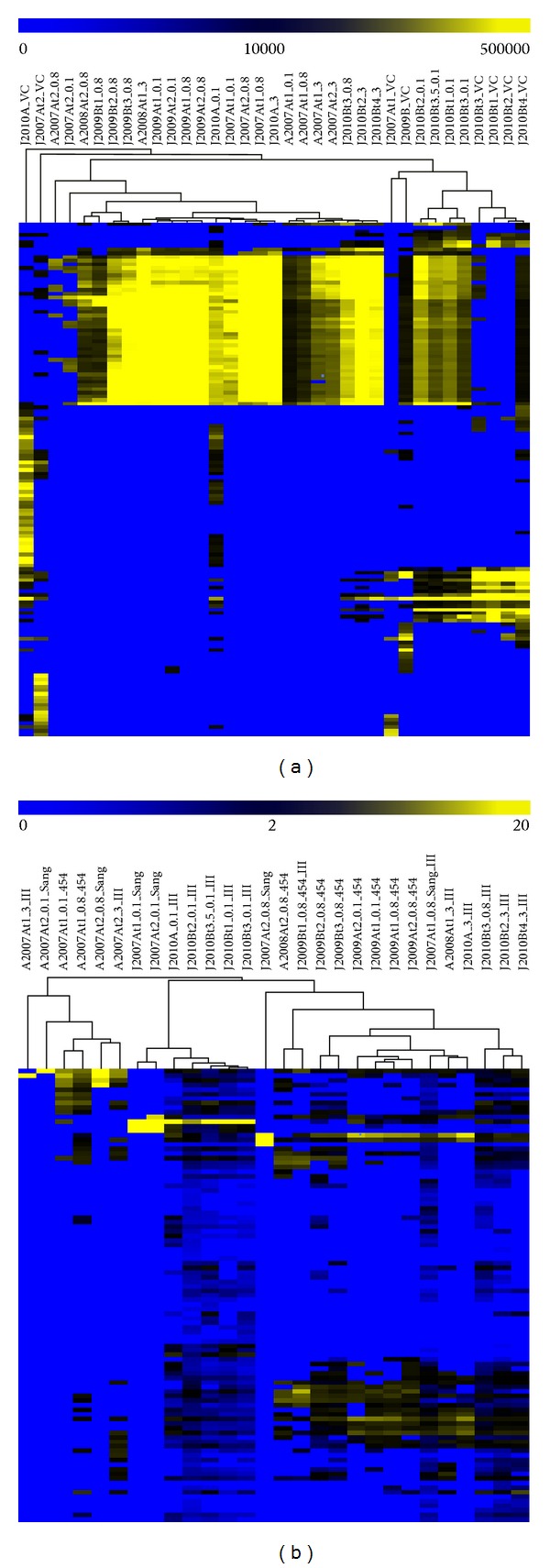


 We used hierarchical clustering (Figure 2(a)) to determine whether patterns in the relative abundances of the 140 viruses (i.e., the 140 viral contigs >10 kb) could be observed, according to season, filter size, and/or sample site. No universal patterns were observed, but there was some clustering according to filter type and/or for samples collected from the same site during the same week. Specifically, all four viral concentrate samples from January 2010, site B clustered together, and they clustered more closely with all of the 0.1 *μ*m filters from the same time series than with other viral concentrate samples. Two additional viral concentrate samples collected during the same season but at different sites and years (J2007At1 and J2009B) complete a larger cluster for that group, indicating similarity among planktonic viral fractions, relative to viruses on larger filter sizes. Interestingly, the remaining two viral concentrate samples (the only sample collected in January 2010 from site A and another sample from site A collected in January 2007) clustered separately from each other and from the rest of the dataset. Overall, this suggests that viral concentrates represent a different part of the viral community than is sampled by other size fractions, particularly fractions >0.8 *μ*m. Interestingly, this is despite the similarity in richness described above (i.e., the number of viral OTUs remains relatively similar across samples, but the composition differs in viral concentrates, relative to host-associated size fractions).

 Although the most obvious trend in the viral assemblage hierarchical clustering analysis was the separation of viral concentrates from other filter sizes described above, some clustering was observed for 0.1, 0.8, and 3.0 *μ*m filter size fractions. Specifically, all four filter samples collected from January 2009 site A cluster together, as do all of the 0.8 and 3.0 *μ*m filters from January 2010 site B, suggesting stability of the active viral and/or proviral assemblages over four days in both cases. Although the 0.1 and 0.8 *μ*m filter samples collected in August 2007 from site A time 1 cluster together, they are separate from filters of the same size collected two days later, suggesting a shift in the active viral assemblage over days or possibly the induction of proviruses on that timescale. Together, these data suggest that, although some turnover in active viruses and/or proviruses was observed, most active viruses and/or proviruses were stable within the archaea-dominated LT system over days.

### 3.2. Archaeal and Bacterial OTUs

**Figure 3:**
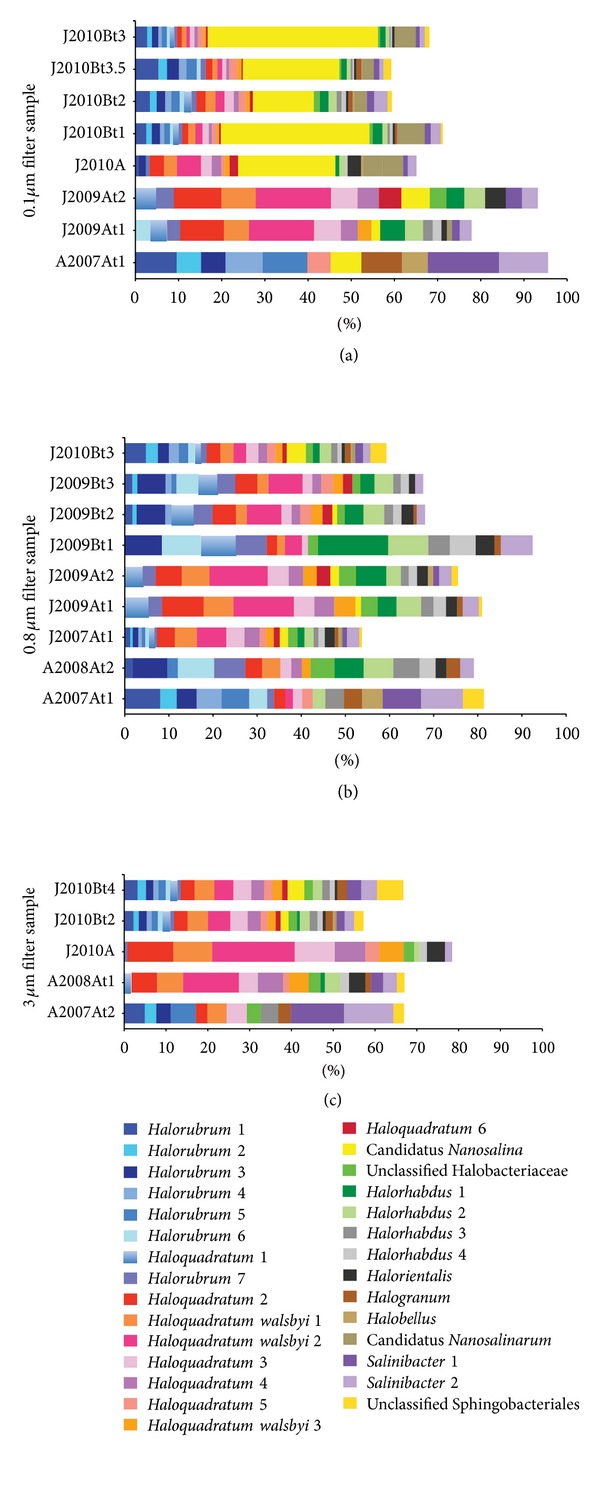


 We also characterized the relative abundances of potential host OTUs across time and between sites in the LT system. Of 101 total archaeal and bacterial 16S rRNA gene OTUs at 97% nt identity, 29 were detected at 5% abundance or higher on any filter in any sample. For easier visualization, Figure 3 shows only those 29 OTUs, and it includes only samples from which at least five of those OTUs were detected. 

 In the filter size-resolved plots (Figures 3(a)–3(c)), it is clear that even the most abundant archaeal groups change significantly over time and space, in terms of both presence/absence and relative abundance. For example, the relative proportions of *Halorubrum*-like and *Haloquadratum*-like OTUs tend to differ significantly across samples, from almost exclusively *Halorubrum*-like organisms (e.g., in the August 2007 time series) to almost exclusively *Haloquadratum*-like organisms (e.g., in the January 2009, site A, time series and in January 2010, site A). Some changes in the relative abundances of these groups can even be observed over hours on 0.8 *μ*m filters from the January 2009, site B, time series (Figure 3(b)). A significant change in temperature was measured between the first and second samples of the January 2009, site B, time series (Table 1), and we hypothesize that this shift in community structure may mark a response to the temperature change. With the exception of the January 2010, site B, time series, the diversity and relative abundance of *Haloquadratum*-like and *Halorubrum*-like organisms are generally anticorrelated, suggesting that these archaea may compete for a similar niche in the LT system. The observation of a relatively low diversity and abundance of *Haloquadratum*-like organisms in some samples (i.e., A2007At1-t2, A2008At2, and J2009Bt1) suggests that *Haloquadratum* species are more dynamic in hypersaline systems than has been previously appreciated [32]. 

 In addition to trends within organism types, specific OTUs also exhibited interesting dynamics. For example, OTU *Haloquadratum walsbyi* 2, which belongs to a species generally considered to be among the most abundant organisms in hypersaline lakes [32], is at relatively low abundance or not detected on 0.8 and 3.0 *μ*m filters from August 2007, site A, and January 2010, site B, though it is at high abundance in that size fraction at site A in January 2009 and January 2010. Interestingly, that OTU also appears dynamic on a days scale in August 2008 at site A, appearing at high abundance on the 3.0 *μ*m filter at time 1 but not detected on the 0.8 *μ*m filter at time 2. Three OTUs, including two related to *Salinibacter*, were more abundant in the August 2007, site A, time series (all filter sizes) than in any other sample. The Nanohaloarchaeon, Candidatus *Nanosalina* [21], was detected at reasonably high abundance on all 0.1 *μ*m filters from January 2010 sites A and B but was less abundant at other sites and times (Figure 3(a)). Consistent with the small cell size for that organism, it and the other abundant Nanohaloarchaeal OTU, Candidatus *Nanosalinarum,* were found at low abundance or not detected on filters larger than 0.1 *μ*m. 

 Overall, analyses of the 29 most abundant archaeal and bacterial OTUs indicate dynamics at the population level across time and space, particularly on months-to-years timescales, with some dynamics indicated over hours to days. These results indicate dynamics in the most abundant archaeal and bacterial populations at the taxon level, in contrast to a previous study, which predicted that the most abundant microbial taxa were stable over timescales of weeks to one month in a hypersaline crystallizer pond near San Diego (SD), CA, USA [6]. That study was based on taxonomic affiliations predicted from ~100 bp metagenomic reads. It is possible that differences in sampling timescales, geochemistry, and/or community composition could result in true differences in microbial dynamics between these two systems. Also, LT microbial eukaryotic communities are dominated by a predatory *Colpodella *sp., and it is unclear what effect top-down grazing may have on shifts in bacterial and archaeal community structure [20]. However, the SD study also suggested stability of viral populations in that system, and we previously demonstrated through a reanalysis of the SD data that the most abundant viral populations from that study were in fact dynamic [8], so it is possible that different sequencing and analytical methods would have revealed dynamics in archaeal and bacterial populations at the SD site as well. Unfortunately, a reanalysis of the existing SD data using the methods in this study is not possible, due to the incompatibility of 454 sequencing reads with the EMIRGE algorithm, which is designed to reconstruct near-complete 16S rRNA genes from paired-end Illumina metagenomic data.

 Based on the relative abundances of the 101 archaeal and bacterial OTUs (including lower abundance LT organisms), we used hierarchical clustering to determine whether similarities in overall archaeal and bacterial community structure would group LT samples according to season, sample type, or filter size (Figure 2(b)). In general, archaeal and bacterial communities sampled from the same location over days clustered more closely than did viral assemblages from the same samples (see above and Figure 2(a)), indicating greater stability of host populations than viral populations over days. The only samples for which no within-time series (i.e., days scale) clustering was observed for host communities were samples from August 2008, site A. For that time series, DNA from different filter sizes was sequenced from each sample, and different sequencing technologies were used (454 and Illumina), so it is possible that similarities between the samples exist but were masked by different methodologies. However, interestingly, all samples and filter sizes from the August 2007, site A, time series clustered together, including DNA from 0.1, 0.8, and 3.0 *μ*m size fractions sequenced by all three technologies (Sanger, 454, and Illumina). This indicates a distinct community at site A in August 2007, which was stable over days and robust to methodological differences in library construction and sequencing.

 In some cases, the archaeal and bacterial communities on the 0.1 *μ*m filters clustered together and separately from other filters from the same time point, reflecting enrichment for Nanohaloarchaea. Specifically, both 0.1 *μ*m filters from January 2007, site A, cluster together, and they belong to a larger cluster that includes all 0.1 *μ*m filters from January 2010, site B. The prevalence of Nanohaloarchaea in these samples but not in others is evident in Figure 3(a), which is based on the 29 most abundant OTUs described above. In all other cases, the 0.1 *μ*m filters clustered with larger filters from the same time series. For the January 2009, site B, single-day time series, from which only DNA from 0.8 *μ*m filters (and a viral concentrate) was sequenced, the morning sample clusters separately from the afternoon and evening samples, which cluster together, indicating a shift in community structure over a single day. This is consistent with the shift in relative abundance of *Halorubrum*-like and *Haloquadratum*-like organisms from that time series (Figure 3) and may be related to a temperature shift, as suggested above.

 Interestingly, a seasonal trend was not indicated in either the analysis of the 29 most abundant OTUs or in the 101 OTU hierarchical clustering analysis (seasonal trends in viral populations would be difficult to infer, as no viral concentrates were sequenced from winter samples). Samples from site A in August 2007 have quite different archaeal and bacterial community structures from samples at the same site in August 2008, and January samples from site A are also fairly distinct year to year. Although some samples from site B, collected in January 2009 and January 2010, might appear to be the exception (Figure 3(b)), there was as much of a shift in community structure over hours in January 2009 at site B as there was across samples collected over years.

 As with any metagenomic study, we cannot say for certain to what extent abundance in metagenomic libraries reflects true abundances. However, we have taken care to avoid biases that are often associated with sequencing-based community analyses. Specifically, by focusing on 16S rRNA gene sequences from metagenomic data, we have avoided PCR amplification biases in our estimates of archaeal and bacterial dynamics. Similarly, we were able to generate enough viral concentrate metagenomic DNA for sequencing without multiple-displacement amplification (MDA), which is known to generate significant biases, especially in viral metagenomes (discussed in [33, 34]).

### 3.3. CRISPR Analyses

 Using the Crass algorithm [30], we identified 549 unique repeats and 8,095 unique spacer sequences from clustered regularly interspaced short palindromic repeat (CRISPR) regions in the LT dataset. Apart from a single sample from January 2009, site A, the only libraries with spacers that matched any of the 140 complete and near-complete viral genomes were those from samples from which viral concentrates were also sequenced (Table 2). This may indicate that different planktonic viral assemblages existed in LT at time points from which viral concentrates were not sequenced, which would be consistent with the viral population dynamics observed across the sequenced viral concentrates [7]. Notably, no spacers from LT August metagenomes matched any of the 140 viruses, consistent with a possible seasonal shift in viral community structure (no viral concentrates were sequenced from August samples), as has been observed in marine systems (e.g., [35]). However, a concomitant seasonal shift in host community structure was not supported, so it is also possible that the August samples could harbor different planktonic viral assemblages each year.

 The only spacer matches to any of the seven previously described complete LT viral and virus-like genomes [7] were to LTV2 (76,716 bp) and LTVLE3 (71,341 bp) in libraries from three samples from the January 2010, site B, time series (Table 2). Of the seven viral concentrate genomes, those two achieved the highest abundance by approximately one order of magnitude [7], and they were at their most abundant in the time series from which spacers targeting them were detected. This indicates that LT CRISPRs can actively target abundant, coexisting viruses, consistent with previous observations of CRISPR sampling of coexisting viruses in an acid-mine drainage system [9, 11]. However, it should be noted that the vast majority of spacers do not match any of the 140 viral contigs > 10 kb, which we assume to be among the most abundant viruses because they assembled significantly. This suggests that most CRISPRs target rare viruses. An alternative explanation might be a preponderance of inactive, vestige CRISPR regions, but if that were the case, then we would expect to see the same CRISPR spacers duplicated across samples (i.e., CRISPR regions would be clonally inherited over multiple generations, as opposed to actively integrating new spacers on subgenerational timescales). Given that only 353 spacer sequences were duplicated across samples, relative to 8,095 unique spacer sequences in the dataset, we infer either that most CRISPR regions were highly dynamic and/or that we detected different CRISPR regions over time, due to shifts in host community structure. Regardless, there is a high diversity of spacers in the LT system, consistent with the high diversity of viruses.

 Of the CRISPR regions that had matches to the 140 LT viral contigs, only five had repeats with matches to assemblies from 0.1, 0.8, and/or 3.0 *μ*m filter sequences (a match would be indicative of the host organism), and none matched any of the 12 complete and near-complete bacterial and archaeal genomes assembled from the LT January 2007 site A time series [36]. Three matches to the January 2010 site B assemblies (Andrade et al., unpublished data) were to contigs that could only be identified at the order level of Halobacteriales, based on best BLAST hits throughout the contigs, and one repeat matched a contig predicted to belong to a Natronomonas-like organism (Table 2). The most interesting CRISPR repeat sequence match was to a contig from a likely Nanohaloarchaeon, based on the taxonomy of best BLAST hits throughout the contig. A spacer associated with that repeat matched LTVLE3, a virus or plasmid described in [7], strongly suggesting that LTVLE3 is a virus or plasmid of the Nanohaloarchaea. Interestingly, while LTVLE3 was only at high abundance in viral concentrates from the January 2010 site B four-day time series, from which the only spacers targeting it were identified, the most abundant Nanohaloarchaeon, Candidatus *Nanosalina* [21], was detected in most LT samples and is as abundant at site A as it is at site B (isolated pools 300 m apart) in 2010 (Figure 3(a)). This suggests that the Nanohaloarchaea at site B may have been adapted locally to the presence of abundant LTVLE3 at site B in January 2010. 

 BLAST searches of all 8,095 LT CRISPR spacers and 549 repeat sequences revealed essentially no hits to the NCBI nr and environmental databases, and no hits were detected for any of the repeat sequences. Three spacers had 100% matches *Homo sapiens*, indicating that they are likely either to be erroneous spacers or representative of mobile genetic elements that are not present in public databases. Overall, these data demonstrate that the LT CRISPR spacers target mobile genetic elements that have not been previously identified, suggesting that LT archaea may be highly adapted to local viral predation, though we cannot rule out the possibility of public database bias (i.e., perhaps LT CRISPRs could target viruses from other locations that are not represented in public databases). In support of local adaptation, despite the relatively small number of spacer matches to the 140 LT viral contigs >10 kb, 1,729 spacers (21%) had matches to unassembled LT viral concentrate reads. This may indicate selection for CRISPR spacers that target locally adapted, potentially coexisting viruses, consistent with previously observed local CRISPR adaptation to coexisting viruses in an acid-mine drainage system and in geographically distinct sludge bioreactors [8, 9]. Because these matches are to unassembled reads, we infer that most targeted LT viruses are at relatively low abundance, at least at the times and locations sampled by this study (with the exception of LTV2 and LTVLE3, as described above). This may indicate successful CRISPR-mediated maintenance of viral populations at abundances low enough to preclude genomic assembly.

**Figure 4:**
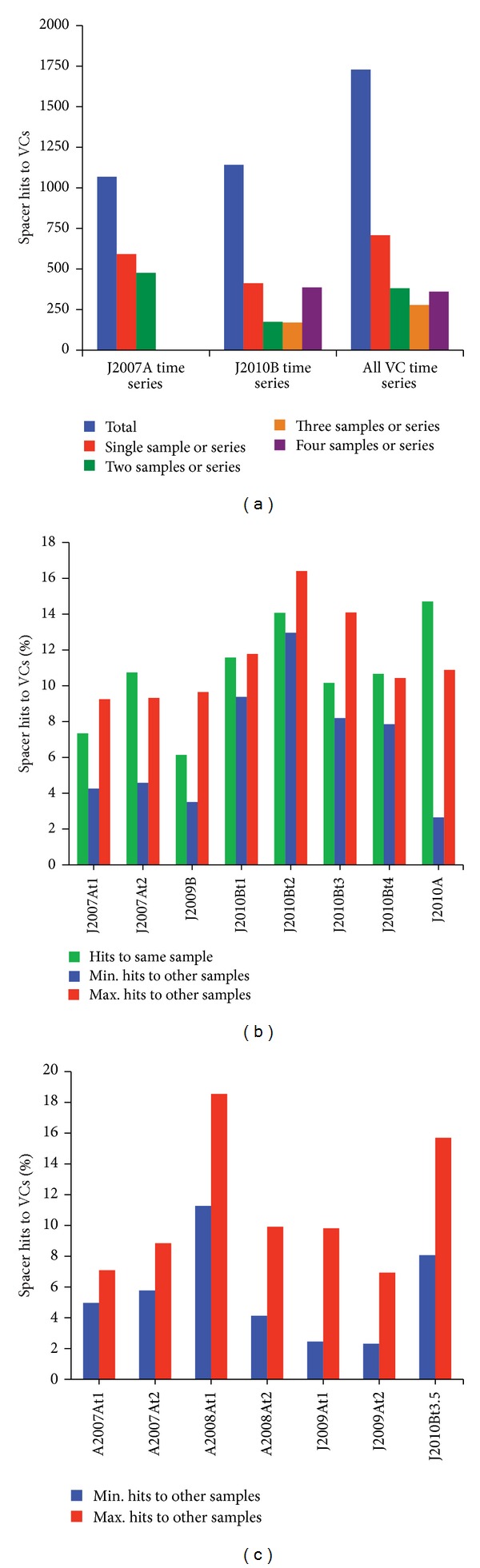


 In terms of the timescales on which CRISPR spacers are retained and match coexisting, low-abundance viruses in the LT system, we found several lines of evidence that support the stability of many CRISPR spacers and low-abundance viruses over all timescales sampled by this study (up to three years). LT spacers tended to match reads from multiple viral concentrate metagenomes collected over the course of days almost as often as (January 2007, site A) or more often than (January 2010, site B) they matched reads from a single viral concentrate metagenome from the same time series (Figure 4(a)). This suggests that many spacers and their targeted, low-abundance viruses are stable over days. On the timescale of years, spacers and their targeted coexisting viruses were found most often across more than one of the four sites and times sampled by viral concentrates (January 2007, site A; January 2009, site B; January 2010, site A; January 2010, site B). Similarly, in all eight samples from which viral concentrates were sequenced, nearly as many (and often more) spacers had hits to viral concentrate reads from other samples as matched viral concentrate reads from the same sample (Figure 4(b)). A similar number of matches to the eight viral concentrates were observed for samples from which viral concentrates were not sequenced (Figure 4(c)). Overall, these results demonstrate that CRISPR regions generally retain spacer targets against low-abundance viruses on timescales of 1–3 years. We also infer that low-abundance viruses are likely more stable in the LT system than their highly dynamic, higher abundance counterparts.

## 4. Conclusions

 Both virus (140 contigs >10 kb) and host (101 16S rRNA gene OTUs) assemblage structures were highly dynamic over time and space in the archaea-dominated LT system, particularly over months to years. However, archaeal and bacterial populations were generally more stable than viral populations, and lower abundance viruses were inferred to be more stable than abundant viruses. Filter size-resolved analyses of viral populations revealed different viral assemblages in the planktonic and host-associated (i.e., active and provirus) fractions, suggesting that a higher diversity of viruses may have the potential to infect than are actively infecting at any given time. Consistent with that hypothesis, CRISPR analyses revealed persistent targeting of lower abundance viral populations, along with a high diversity of spacer sequences. However, interestingly, the most abundant viruses (~2% of the viral community, relative to ≤0.1% for most viruses, [7]) were also targeted by CRISPRs, indicating that archaeal hosts strike a balance between protection against persistent, low-abundance viruses and those viruses potentially abundant enough to effect catastrophic changes in host community structure.

## Supplementary Material

Table S1 reports the relative abundances of 16S rRNA gene OTUs across samples. The sequence IDs for each OTU appear in rows, and sample IDs are in columns. Values throughout the table indicate the relative abundance of a given OTU in a given sample, calculated as described in the Materials and Methods section.

## Figures and Tables

**Table 1:** Sequencing and sample information, all filter sizes and viral concentrates.

Sample	Date	Time	*T* (°C)	pH	TDS (wt%)	Filter size	Sequencing technology	Library type(s)	Reads
J2007At1	Jan. 23, 2007	15:00	22	7.2	31	0.1	Sanger	8–10 kb	650566
						0.8	Sanger, Illumina	fosmid, 8–10 kb, and 100 cycles SR	22192398
						VC	Illumina	100 cycles PE	2436330

J2007At2	Jan. 25, 2007	15:00	28	7.1	31	0.1	Sanger	8–10 kb	905142
						0.8	Sanger	fosmid, 8–10 kb	832880
						VC	Illumina	100 cycles PE	7330099

A2007At1	Aug. 7, 2007	14:00	24	nm	25	0.1	454	SR	5558982
						0.8	454	SR	5333532
						3	Illumina	100 cycles SR	1297810

A2007At2	Aug. 9, 2007	10:30	23	nm	25	0.1	Sanger	8–10 kb	12609766
						0.8	Sanger	8–10 kb	746394
						3	Illumina	100 cycles SR	3949427

A2008At1	Aug. 11, 2008	11:00	12	7.2	25	3	Illumina	100 cycles SR	15786056

A2008At2	Aug. 12, 2008	10:45	11	nm	25	0.8	454	SR and PE	10359280

J2009At1	Jan. 3, 2009	11:45	20	7	28	0.1	454	SR and PE	6303283
						0.8	454	SR	5920276

J2009At2	Jan. 7, 2009	15:00	27	6.9	27	0.1	454	SR	5519946
						0.8	454	SR	6181544

J2009Bt1	Jan. 5, 2009	7:21	18	6.9	24	0.8	454, Illumina	SR, 100 cycles SR	6844436
J2009Bt2	Jan. 5, 2009	12:37	30	7.1	26	0.8	454	SR	7372159
J2009Bt3	Jan. 5, 2009	18:00	36	7	27	0.8	454	SR	7546428
J2009B*	Jan. 5, 2009					VC	Illumina	100 cycles PE	19567468

J2010Bt1	Jan. 7, 2010	7:45	20	7.2	32	0.1	Illumina	100 cycles PE	13213244
						VC	454	SR	2373021

J2010Bt2	Jan. 7, 2010	20:00	32	7.3	36	0.1	Illumina	100 cycles PE	27300634
						3	Illumina	100 cycles PE	23375315
						VC	Illumina	100 cycles PE	3312787

J2010Bt3	Jan. 8, 2010	8:00	21	7.2	34	0.1	Illumina	100 cycles PE	38287968
						0.8	Illumina	100 cycles PE	13808599
						VC	454	SR	2243916

J2010Bt3.5**	Jan. 9, 2010	16:15	45	7.1	27	0.1	Illumina	100 cycles PE	21747692

J2010Bt4**	Jan. 10, 2010	12:50	33	7.2	32	3	Illumina	100 cycles PE	15465664
						VC	Illumina	100 cycles PE	9610233

J2010A	Jan. 10, 2010	12:50	37	7.1	35	0.1	Illumina	100 cycles PE	52520328
						3	Illumina	100 cycles PE	6854358
						VC	Illumina	100 cycles PE	9268384

VC: viral concentrate.

Nm: not measured.

PE: paired-end sequencing.

SR: single-read sequencing.

*VC from J2009B is a pool of DNA from three viral concentrates collected throughout a single day.

**To maintain consistent sample naming with previous publications, we are retaining sample names that were based on consecutive viral concentrate samples. Sample 3.5 was actually collected fourth in the series and sample 4 was fifth.

**Table 2:** CRISPR analyses by sample.

Sample	Unique repeats	Unique spacers	Hits to 140 viruses	Viral contig match(es)	Predicted host
J2007At1	67	681	1	scaffold_16	

J2007At2	82	633	1	Contig999004	

A2007At1	30	141	0		

A2007At2	64	554	0		

A2008At1	20	275	0		

A2008At2	23	121	0		

J2009At1	41	163	1	scaffold_117	

J2009At2	40	173	0		

J2009B	29	114	0		

J2010Bt1	22	501	0		

J2010Bt2	79	1798	4	LTV2	
				LTV2	
				LTVLE3	
				Contig999004	

J2010Bt3	58	1171	8	LTVLE3	
				Contig1100059	Natronomonas-like
				Contig998975	
				scaffold_55	
				scaffold_29	
				LTVLE3	
				LTVLE3	Nanohaloarchaea
				LTVLE3	Nanohaloarchaea

J2010Bt3.5	60	930	4	LTV2	
				LTVLE3	Nanohaloarchaea
				Contig999004	
				Contig998975	

J2010Bt4	43	853	1	Contig999004	

J2010A	20	340	0		
