# Supplementary material for: Virus-Host and CRISPR Dynamics in Archaea-Dominated Hypersaline Lake Tyrrell, Victoria, Australia
Source: Archaea. 2013 Jun 18;2013:370871. doi: 10.1155/2013/370871 (PMC3703381; doi:10.1155/2013/370871)
Supplement: Supplementary file 1 — Table S1 reports the relative abundances of 16S rRNA gene OTUs across samples. The sequence IDs for each OTU appear in rows, and sample IDs are in columns. Values throughout the table indicate the relative abundance of a given OTU in a given sample, calculated as described in the Materials and Methods section. [file 370871.f1.pdf]

OTU_SequenceID	A2007At1_0.1	A2007At1_0.8	A2007At1_3.0	A2007At2_0.1
1|GL982576.1	7.130537624	0	0	0
74|AF435111.1.1471	9.594441433	8.03533073	0	0
21|FN391220.1.1345	0	2.09553546	0	0
184|AY838278.1.1473	0	1.78015737	0	0
36|CU467230.3.1357	0	0	0	0
268|FN391240.1.1345	0	0	0	0
2|GL982569.1	0	0	0	0
638|AJ270249.1.1461_m01	5.654659242	4.5001624	0	0
26|CU467268.2.1356	0	2.82582957	0	0
17|FN393543.1.1362	11.32410191	9.52735198	0	100
20|CU467211.3.1344	0	2.50940372	0	0
564|AY862784.1.1402	0	4.71076372	0	0
176|CU467143.1.1344	10.32868477	6.25412559	0	0
112|FN391263.1.1344	0	4.08315085	0	0
254|FN391293.1.1345	0	0	0	0
107|FN391274.1.1344	0	0	0	0
211|EF459716.1.1519	16.48598039	8.68913779	100	0
179|EF533958.1.1551	0	0	0	0
643|EF468473.1.1470_m01	0	3.56241028	0	0
482|GQ282618.1.1471	0	2.30299347	0	0
839|FN393543.1.1362_m76	0	2.61732379	0	0
140|EF690618.1.1445	0	1.5716516	0	0
88|AM947502.1.1345	0	0	0	0
98|CU467180.3.1354	0	1.36209805	0	0
289|DQ432495.1.1399	9.350514956	4.08315085	0	0
316|EF690563.1.1439	0	4.18687985	0	0
22|EF459702.1.1440	0	0	0	0
101|GQ282622.1.1467	0	0	0	0
58|AM947497.1.1343	0	0	0	0
30|CU467261.1.1348	0	0	0	0
287|EU562182.1.1478	8.616271621	5.65165914	0	0
65|DQ432537.1.1395	0	0	0	0
59|AM947487.1.1342	0	1.56746053	0	0
44|AM947477.1.1344	0	0	0	0
842|EF468473.1.1470_m34	0	0	0	0
76|EU722666.1.1246	0	0	0	0
163|EF690613.1.1440	0	0	0	0
69|AM947448.1.1343	0	0	0	0
199|AM947454.1.1343	0	0	0	0
41|CU467267.2.1356	4.422707338	3.01337999	0	0
187|EF690583.1.1440	0	0	0	0
154|CU467189.3.1357	6.026708717	4.70866818	0	0
216|AY987828.1.1441	0	0	0	0
841|EF459716.1.1519_m39	0	0	0	0
840|FN391274.1.1344_m38	0	0	0	0
19|EU722669.1.1444	0	0	0	0
14|EF690585.1.1438	0	0	0	0
138|FJ429313.1.1489	0	0	0	0

133|EU931577.1.1473	0	0	0	0
208|AM947451.1.1344	0	0	0	0
82|AM947499.1.1344	0	0	0	0
222|AY498650.1.1371	0	0	0	0
180|FN391283.1.1344	0	0	0	0
173|EF690565.1.1442	0	0	0	0
39|AM947450.1.1345	0	0	0	0
34|GQ282621.1.1473	0	0	0	0
200|FN391291.1.1344	0	0	0	0
23|AM947468.1.1342	0	0	0	0
565|AM947493.1.1343	0	0	0	0
114|AM947496.1.1351	0	2.50835595	0	0
177|CU467118.3.1354	0	0	0	0
96|FN391257.1.1346	0	0	0	0
115|AM947444.1.1348	0	0	0	0
130|CU467153.1.1343	0	0	0	0
132|FN391289.1.1340	0	0	0	0
24|CU467116.3.1355	0	0	0	0
93|FJ172059.1.1341	5.649731434	3.76777276	0	0
265|CU467142.1.1344	0	0	0	0
54|AM947441.1.1346	0	0	0	0
87|AM947456.1.1341	0	0	0	0
61|CU467214.1.1346	0	0	0	0
44|AM180088.1772798.1774270	0	0	0	0
47|FN391220.1.1345	0	0	0	0
32|CU467207.1.1346	0	0	0	0
103|CU467197.3.1345	0	0	0	0
38|AM176545.1.1353	0	0	0	0
26|AM947443.1.1341	0	0	0	0
54|CU467264.1.1344	0	0	0	0
40|CU467180.3.1354	0	0	0	0
30|CU467114.3.1355	0	0	0	0
58|CU467266.1.1348	0	0	0	0
35|AM947446.1.1344	0	0	0	0
51|CU467178.3.1354	0	1.6753806	0	0
76|AM947441.1.1346	0	0	0	0
20|GL982569.1	0	0	0	0
27|AM180088.1772798.1774270	0	0	0	0
525|AM180088.1772798.1774270_m0	0	0	0	0
54|CU467211.3.1344	0	0	0	0
535|AM180088.1772798.1774270_m0	0	0	0	0
563|AM180088.1772798.1774270_m3	0	0	0	0
18|CU467207.1.1346	5.415660573	2.40986578	0	0
11|CU467114.3.1355	0	0	0	0
36|AM947441.1.1346	0	0	0	0
253|CU467207.1.1346_m37	0	0	0	0
3|AM947487.1.1342	0	0	0	0
136|AM947450.1.1345	0	0	0	0
6|FN391234.1.1344	0	0	0	0

431|GL982576.1_m20	0	0	0	0
54|FN393470.1.1364	0	0	0	0
59|AM947441.1.1346	0	0	0	0
95|AM947442.1.1345	0	0	0	0

A2007At2_0.8	A2007At2_3.0	A2008At1_3.0	A2008At2_0.8	J2007At1_0.1	J2007At1_0.8
0	0	0	0	52.0387008	1.87915656
0	4.94526494	0	1.76175739	0	1.26343407
0	4.85517731	4.52655466	2.46674234	0	4.0990371
0	0	13.3753788	0	0	6.69284036
0	3.73317682	3.00164295	0	0	1.14246698
0	0	1.76918667	0	0	1.27687486
0	0	0	0	47.9612992	0.36263246
0	3.38647594	0	7.89019154	0	1.24932124
0	0	3.77493941	6.68960218	0	2.11020371
33.2665777	11.8301439	3.29843462	3.1005238	0	2.79554949
0	2.80363626	6.12180985	3.80057386	0	4.08546191
0	2.50607409	1.8043584	0	0	0.57795388
16.6889186	6.04542601	0	2.39271892	0	0.7795657
0	0	0	8.22717435	0	0.80644728
0	0	1.01082615	6.61205383	0	1.54179279
0	0	0	0	0	0
28.9052069	12.7228304	2.88456442	0	0	1.04717179
0	0	1.59959143	0	0	1.03494067
0	3.49840299	0	3.92535619	0	1.07485981
0	0	3.82071085	0	0	2.12256923
0	4.25186318	0	0	0	0.85994161
0	0	0	7.04420961	0	0.5766098
0	0	0	0	0	0
0	0	0	0	0	0.85994161
21.1392968	3.00565095	1.37507046	3.09981882	0	0.80644728
0	4.16860037	0	5.97968233	0	1.04838146
0	0	0	0	0	0
0	0	0	0	0	0
0	0	3.90261764	2.46744732	0	2.24353633
0	3.32914744	2.85035629	5.42344921	0	2.15052607
0	0	0	0	0	0.80644728
0	0	0	0	0	0.49730915
0	0	0	0	0	0.84663523
0	0	0	0	0	0.41666443
0	0	0	0	0	0
0	0	0	0	0	0.59139467
0	0	0	0	0	0
0	2.90054872	1.59140075	2.60350942	0	1.30375643
0	0	0	0	0	0.68548019
0	0	0	2.04375137	0	0.40295482
0	0	0	0	0	0.32257891
0	0	0	0	0	0.56451309
0	0	0	4.36667677	0	0.51074994
0	4.94935983	0	0	0	0
0	0	0	0	0	0
0	0	0	0	0	0.34946049
0	0	0	0	0	0.61827625
0	0	0	0	0	0.64515782

0	2.93467282	2.03321561	0	0	0.91397358
0	0	0	0	0	0.41666443
0	0	0	0	0	0.41666443
0	0	0	0	0	0.71236176
0	0	0	2.18333839	0	0
0	0	0	0	0	0.80496879
0	0	0	0	0	1.35738518
0	0	0	0	0	0
0	0	0	0	0	0
0	0	0	0	0	0.36276687
0	0	0	0	0	0
0	0	0	0	0	0.80644728
0	0	2.56850058	0	0	1.08615007
0	0	0	0	0	0
0	0	0	0	0	0.43010521
0	0	0	0	0	0
0	0	0	0	0	0
0	0	0	0	0	0.75174327
0	2.75859245	0	0	0	0.57768507
0	0	0	0	0	0
0	0	0	0	0	1.85469433
0	0	0	0	0	0
0	0	0	0	0	0
0	0	6.04134847	2.39483387	0	3.45791152
0	0	3.66556976	0	0	2.23103639
0	0	4.69277727	2.00427221	0	1.76074322
0	0	2.2707453	0	0	1.97552701
0	2.47058502	4.74192134	0	0	2.08049957
0	0	3.47670234	0	0	2.34918092
0	0	2.0004529	3.59401327	0	1.46504589
0	0	0	0	0	1.69340487
0	0	0	0	0	1.62526008
0	0	0	0	0	1.65321692
0	0	0	0	0	1.10147257
0	0	0	0	0	1.19623013
0	0	0	0	0	1.29018124
0	0	0	0	0	0.26881576
0	4.57945456	6.21431634	4.15518129	0	4.93236596
0	2.50743905	0	0	0	0.73924334
0	3.24861456	1.63669039	0	0	1.54569061
0	0	0	4.50626379	0	0
0	0	0	0	0	0
0	0	1.40831498	0	0	1.42472352
0	2.56886244	2.54200132	1.26685795	0	1.58574416
0	0	0	0	0	0
0	0	0	0	0	0
0	0	0	0	0	0.41666443
0	0	0	0	0	0.71222735
0	0	0	0	0	0.38964844

0	0	0	0	0	0
0	0	0	0	0	0
0	0	0	0	0	0.56451309
0	0	0	0	0	0

J2007At2_0.1	J2007At2_0.8	J2009At1_0.1	J2009At1_0.8	J2009At2_0.1	J2009At2_0.8
36.2557563	0	2.03469637	1.2757077	6.56309647	1.95767479
0	0	0	0	0	0
0	35.8490566	6.24153306	4.64236427	6.17431709	4.74510433
0	39.6226415	15.0727993	13.6854152	17.4090241	13.2172746
0	0	2.68048559	2.31983574	1.56137551	2.31285971
0	0	3.67744736	5.37816823	4.8452705	4.09294204
47.8126107	0	1.18372769	0	0	0
0	0	0	0	0	0
0	0	4.20486381	5.56397051	4.82336744	3.26021751
0	0	2.69429575	3.63122286	3.67032761	2.84979479
0	0	10.1182412	9.41667508	11.0102006	5.93182588
0	0	0	0.73916995	0	1.42370948
0	0	0	0	0	0
0	0	3.66824059	0	0	0
0	0	5.71806237	4.30374634	4.11855815	6.88155949
0	0	0	0	0	0
0	0	1.83609317	0	3.67032761	1.30432458
0	0	0	0	0	0
0	0	0	0	0	0
0	0	3.47818653	3.67363425	0	4.27231636
0	0	1.37904276	0	0	0
0	0	3.07374624	3.07516241	4.1177759	2.90800235
0	0	0	0	0	0
0	0	0	0	0	0
0	0	0	1.27840048	0	0
0	0	2.20962502	2.82338685	0	1.77830046
0	0	0	0	0	0
0	0	0	0	0	0
15.931633	0	1.24685983	2.48813491	4.83979474	2.43224461
0	0	0	3.76720859	3.88622923	3.85476619
0	0	0	0	0	0
0	0	0	1.8829311	0	2.01469444
0	0	0	0	0	1.00734722
0	0	0	0	0	0
0	0	0	0	0	0
0	0	0	0	0	0
0	0	0	0	0	0
0	0	2.82582105	2.68740112	1.63881849	0
0	0	0	0	0	0
0	0	0	0	0	0
0	0	0	0	0	0
0	0	0	0	0	1.24730493
0	0	0	0	0	0
0	0	0	0	0	0
0	0	0	0	0	0
0	0	0	0	0	0
0	0	0	0	0	0
0	0	0	0	0	0

0	0	1.49610027	0	0	0
0	0	0	0	0	0
0	0	0	0	0	0
0	0	0	1.41371302	0	0
0	0	0	0	0	0
0	0	0	0	0	0
0	0	0	0	5.22700961	3.08500086
0	0	0	0	0	0
0	0	0	0	0	0
0	0	0	0	0	0
0	0	0	0	0	0
0	24.5283019	0	0	0	0
0	0	0	0	0	0
0	0	0	0	0	0
0	0	0	0	0	0
0	0	0	0	0	0
0	0	0	0	0	0
0	0	0	0	0	0
0	0	0	0	0	0
0	0	0	0	0	0
0	0	0	0	0	3.14558424
0	0	0	0	0	0
0	0	0	0	0	0
0	0	3.91024714	4.50301256	4.91019744	3.26437519
0	0	3.61299996	0	0	3.08440691
0	0	3.15529192	4.77565721	0	3.08559482
0	0	0	0	0	2.54984765
0	0	0	0	0	0
0	0	4.40215175	4.3717392	3.59366689	4.15174355
0	0	2.03469637	2.82338685	0	1.89768536
0	0	0	0	0	0
0	0	0	0	0	0
0	0	0	0	0	0
0	0	0	0	0	0
0	0	0	0	0	0
0	0	0	0	0	0
0	0	0	0	0	0
0	0	5.78119451	6.79053485	7.9406427	6.28344708
0	0	0	0	0	0
0	0	2.26355039	2.68942071	0	1.9600506
0	0	0	0	0	0
0	0	0	0	0	0
0	0	0	0	0	0
0	0	0	0	0	0
0	0	0	0	0	0
0	0	0	0	0	0
0	0	0	0	0	0
0	0	0	0	0	0
0	0	0	0	0	0

0	0	0	0	0	0
0	0	0	0	0	0
0	0	0	0	0	0
0	0	0	0	0	0

J2009Bt1_0.8	J2009Bt2_0.8	J2009Bt3_0.8	J2010A_0.1_1	J2010A_3.0_1	J2010Bt1_0.1
0	1.08716364	0	22.5470678	0	34.3727114
0	1.79118292	1.72408034	0.73282856	0	2.57444435
1.32909704	2.34548325	2.18891957	2.47094758	9.62203334	1.42240989
3.87936262	7.84595282	7.79426828	5.60867519	19.6878154	1.66927442
0	0.98576309	0.91715285	0.52575751	2.30798114	0.58777268
7.92486236	5.030216	4.2706756	0	0	1.15203446
0	0	0	9.28634712	0	6.37039789
8.42448643	6.52638456	6.38165806	1.68155968	0	1.93964985
9.07023841	4.68960542	4.21945978	1.39989045	1.35806623	1.21081173
7.20695593	1.83711941	1.77947136	1.97281206	1.70867651	2.29231346
2.36290655	5.37218767	5.00801639	3.29763456	10.9961916	1.38714353
0	0	0	0	0	0.44670724
0	0	1.11227398	0.40399523	0	1.36363262
8.80951226	0	4.97711989	0	0	0.83463721
14.9426402	4.23466402	3.21629776	0.88315237	0	2.24529165
0	0	0	1.53123588	0	2.59795526
0	0	0	1.09877308	0	1.3851451
0	0	0	0	0	0
0	3.0382736	3.00698094	0.8173857	0	1.6222526
0	3.16383335	3.85148526	1.56900473	4.69057058	0.90505238
1.69653901	0	0.86204017	0	0	0.79937085
7.01899057	4.25133726	4.10422419	0.51664413	0.79923539	0.83463721
0	0	0	0.45097142	0	0
0	0	0	1.05226665	0	0.45846269
1.45339672	0.74655306	0	0	0	0.56426178
4.89558827	2.00555321	1.74439966	0	0	0.54075087
0	0	0	0	0	0.74059358
0	0	0	0	0	0.41144088
4.24074119	2.72046113	1.44629019	3.12786261	4.2965607	0.48185605
3.24209939	1.73367723	1.79366705	0	2.51571407	0.76410449
0	1.36006043	1.36306449	0	0	0.94043629
0	0	0.66719738	0	0	1.140279
0	0.83740523	0	1.62537616	0	0.3291527
0	0.71354684	0	0.52613332	0	0
0	0	1.7808631	0	0	0
0	0	0	0	0	0.89341448
0	0	0	0	0	0.43495179
0	1.3168461	0	0.90194284	3.28276548	0.54075087
0	0	0	1.96360473	0	0.22335362
0	0	0	0	0	0.74059358
0	0	0.86259686	0	0	0.95219175
0	0	0	0.45097142	0	0
4.72823846	0	2.69745925	0	0	0.61128359
0	0	0	0	0	0
0	0	0	0	0	0
0	0	0	0	0	0
0	0	0	0	0	0
0	0	0	0	0	0

0	1.05415742	0.84728779	0	0	0.38792997
0	0	0	0.68585237	0	0
0	0	0	0	0	0
0	0	0	0	0	0
0	0	0	0	0	0.3291527
0	0.95173606	0	0	0	0
0	2.21039594	2.13464193	1.96351077	0	0.39968542
0	0	0	0	0	0
0	0	0	0	0	0
0	0	0	0	0	0
0	0	0	0	0	0.36441906
0	0	0	0	0	0.21159817
0	0	0	0	0	0
0	0	0	0	0	0
0	0	0	0	0	0.30564179
0	0	0	0	0	0
0	0	0	0	0	0
0	0	0	0	0	0
0	0.91736876	1.15430436	0	0	1.25783354
0	0	0	0	0	0
0	0	1.69040037	1.75662764	0	0.52899541
0	0	0	0	0	0.27037543
0	0	0	0	0	0
0	1.89734725	2.04417921	2.16081077	7.31697802	0.72883813
0	2.67520518	2.88450833	1.3526324	0	0.69357177
0	2.50677138	2.21202236	1.18379998	5.8316388	0.54075087
0	2.04060105	2.28216576	0.90194284	3.14525213	1.1167681
0	2.14234188	2.58500713	0	0	0.62303904
0	2.17568837	2.56858466	1.23077617	4.45016604	0.79937085
5.86573209	1.93103401	1.80146076	0.53552856	1.69014634	0.6583054
0	0	0	1.21189174	0	0
0	0	0	0	0	0
0	1.80241184	0	0	0	0
0	1.02013039	1.00093525	0.55422509	0	0
0	0.98610336	0	0.86436189	0	0
0	0	0	0	0	0
0	0	0	1.65346792	0	2.01018257
1.757173	2.4148984	2.44666874	3.00638219	9.29092851	1.64576351
0	0	1.05660461	0	0	0
0	1.29200637	1.55039191	0.91124413	3.68701572	1.1167681
0	2.60715112	0	0	0	0.98745811
0	0	0	0	0	0
0	2.58163085	2.69662421	0.78919999	3.322264	1.15203446
1.15143945	1.69794885	1.27455019	1.21179779	0	0.90516993
0	0	0	0	0	0
0	0	0	0	0	0
0	1.46180125	0	1.39942069	0	0.54075087
0	0	0	1.02389303	0	0
0	0	0	1.31523936	0	0.16457635

0	0	0	4.77268692	0	3.17385493
0	0	0	1.79420859	0	0
0	0	0	0.87366318	0	0.30564179
0	0	0	0.40399523	0	0

J2010Bt2_0.1_	J2010Bt2_3.0_	J2010Bt3.5_0_	J2010Bt3_0.1_	J2010Bt3_0.8_	J2010Bt4_3.0_
14.1034636	1.87367022	22.3450606	39.4244056	4.32184865	4.01952605
3.42478541	2.23646442	5.36989457	2.75681771	4.84546303	3.18673433
2.13458213	4.12844355	0.94506188	1.07342641	2.76318564	4.30793011
2.10485258	5.39054392	1.09521189	0.77508299	2.88118878	4.54854853
1.02864264	2.6568753	0.90090011	0.67972237	1.92485256	2.97454147
1.53999101	1.64467861	0	0.83861438	1.36207843	1.55264949
2.91302083	0.34951807	2.68468231	4.92895602	0.58936086	0
1.98587489	1.87453794	2.76426182	1.43568241	2.51447536	1.72704407
1.89674568	2.35464803	0.86539404	0.86402694	2.60589178	2.25007414
3.0680902	2.28939541	0.998056	1.14979114	1.66330731	3.80349189
2.12269031	3.26948641	1.53682959	1.12444211	3.09073931	3.31795281
1.01675082	2.18648368	1.77530315	1.06097425	3.61448466	6.2901895
1.89074031	1.46514711	2.40231196	1.21338606	2.134796	1.78328055
1.20696052	1.34002173	0.89206775	0.82571751	1.51911035	1.19971175
2.00377209	0.6767357	1.30710006	1.28320706	1.54543514	0
0.8443194	0.1238238	1.28934703	2.02588904	0	0
1.68697394	1.91184995	1.25322271	0.92063341	1.75406888	3.4173654
1.84905948	1.80538057	3.00264706	1.60734423	2.72415685	0
1.63488775	1.20431014	1.978094	1.03530757	2.09511236	1.3192527
1.15945269	2.24488131	1.04204112	0.8194279	1.51911035	1.90358976
0.87999486	0.84559423	0.31796474	0.66047236	0.78581448	1.45753915
1.14756087	0.90937174	1.18344711	0.64789314	1.36194746	0.81850211
0	1.18704249	2.44620876	0.96567717	0	1.64361124
0.71945526	1.41013359	0.90081178	0.48912819	1.03465573	1.30158277
1.01669136	1.73422743	1.17461475	0	1.33588461	2.28802608
1.10593949	1.84555605	0.42395299	0.29853401	1.427432	1.80233335
0.67188797	0.34370434	0.5564383	0.43201347	0.55007013	0
0.5826993	0	0	0.42566033	0	0
0.54702383	0.65374109	0.55626165	0.55272311	0.73342685	0.59893397
1.4270187	2.02231085	0.45045005	0.49554486	1.59782277	2.02589644
1.43296461	1.19710806	1.73078808	0.79401534	2.22608144	1.24181229
0.8264222	0.36166616	0.52994124	0.5399533	0	0
0.45188925	0	0.37961457	0.17153476	0.45839178	0
0.79675211	0	0.92739717	0.36841854	1.13916905	0
1.05242629	0.76177237	1.11278828	0.93372087	1.24407529	0
0.79669265	0	0.49461182	0.48906466	0.56316704	0
0.38053832	0	0.72407638	0.57813567	0.62865158	0
0.8978326	1.33906723	0.47694711	0	1.12633408	1.472597
0.22594463	0	0.30030004	0.41295405	0	0
0.41621379	0.14794645	0.30913239	0.43201347	0.19645362	0
0.92161624	0.73643492	1.28033803	0.83855085	1.1000093	1.05220621
0.36270059	0.59291384	0.16781473	0	0.40600415	0
0.87987594	1.00325916	0.68865864	0.4636521	1.06071857	0.9985818
0.58858575	0.65261306	0	0	0	0
0.24378236	0	0.8918911	0.90837185	0	0
0.28540374	0	0	0	0	0
0.32096029	0	0	0	0	0
0.19621507	0	0.5564383	0	0	0

0.7432389	1.36735494	0.54760595	0.27953813	0.73342685	1.14547273
0.37459241	0	0	0	0	0
0.273393	0.45026049	0	0	0	0.61706485
0.41615433	0.80498488	0.23847356	0	0	1.09200197
0.40420305	0.46379694	0	0	0.64174849	0
0.72540117	0.56818379	1.37784722	0.501898	1.19181862	0
0.70756344	1.19780224	0.282547	0.42566033	0.98200616	1.27054514
0.46372162	0	0	0.62877019	0	0
0.29129019	0.1593136	0	0	0	0
0.24378236	0	0	0.22871301	0.26193816	0
0	0	0.23838523	0.34306952	0	0
0.21999872	0	0.22080885	0.26664125	0.35361651	0
0.4221597	1.12057506	0	0	0.47135772	1.3573583
0	0	0.38862357	0	0	0
0.40432196	0	0.30913239	0	0.43206699	0
0.26156064	0	0.30913239	0	0	0
0	0	0	0	0	0
0.29729556	0.84533392	0	0	0	0.62874238
1.53999101	1.27424847	1.9957587	1.13079525	2.65841038	2.04372003
0.19026916	0	0	0	0	0
0.67176905	1.41533992	0.42395299	0.24141929	1.07394645	0
0.13675596	0	0	0	0	0
0.30918738	0.61972642	0	0	0.51077941	0.78592797
1.1653986	3.10114852	0.70649999	0.77508299	2.00382692	3.03830688
0.86804358	2.15272933	0.57392636	0.33036324	1.19168765	1.83275637
0.94539989	1.87818237	1.02455306	0.62254411	1.4926546	2.27404379
1.29614919	1.95627727	1.14802937	0.74331729	1.78091754	1.96074816
0.78486028	1.74446654	0.78599118	0.46371563	1.27040007	0
1.18918225	2.30432021	0.75075009	0.62889725	1.59756083	2.19568147
1.04053447	1.29568118	0.37087054	0.34935913	0.95581234	1.20309208
0.49351063	1.02798921	0	0.25412557	0	0
0.60648295	0	0	0	0.91678356	0
0.51134837	0.96230272	0.28245868	0	0.69413612	0
0.52324019	0.67699602	0.30030004	0.22864948	0.58936086	0
0.35080876	0	0.33562945	0.2794746	0.44529487	0
0.4756729	0	0.41512064	0	0.78581448	0
0.7432389	0	0.79464689	1.07317228	0	0
2.29512174	4.81906279	1.15695005	1.11179937	2.94667332	4.70711699
0.75513073	1.36197507	1.04204112	0.67343276	1.10014027	1.33676898
1.51620736	2.06517627	1.74871776	1.08625975	2.29195889	2.36869318
1.08810176	0.72810479	1.20111182	0.87666968	1.12620311	0
0	0	1.12162063	0.79376122	0	0
1.59350421	1.71904231	1.82812063	1.22609234	1.95117735	2.05893154
1.01675082	1.70186144	1.17470308	0.73048395	1.9776331	2.0171383
0	0	0	0	0	1.05819862
0	0.75621896	0	0	0	0
0.70161753	0	0.68009126	0.46377916	0.53684226	0
0.33891694	0	0	0	0	0
0.20216098	0	0.15898237	0	0	0

1.25458727	0	2.54371794	4.24376995	0	0
0	0	0	0.89579263	0	0
0.35663576	0.69018538	0.4945235	0	0.58922989	0.70418532
0	0	0	0	0	0

_16S
